# Exploring the importance of predicted camel *NRAP* exon 4 for environmental adaptation using a mouse model

**DOI:** 10.1111/age.13490

**Published:** 2024-10-30

**Authors:** Sung‐Yeon Lee, Bo‐Young Lee, Byeonghwi Lim, Rasel Uzzaman, Goo Jang, Kwan‐Suk Kim

**Affiliations:** ^1^ Department of Animal Sciences Chungbuk National University Cheongju South Korea; ^2^ GEM Division Macrogen Inc. Seoul South Korea; ^3^ Laboratory of Theriogenology, College of Veterinary Medicine Seoul National University Seoul South Korea; ^4^ Department of Biological Science University of New Hampshire Durham New Hampshire USA; ^5^ Department of Animal Science and Technology Chung‐Ang University Anseong South Korea

**Keywords:** camel, CRISPR/Cas9, environment adaptation, knock‐in, mouse, *NRAP*

## Abstract

Camels possess exceptional adaptability, allowing them to withstand extreme temperatures in desert environments. They conserve water by reducing their metabolic rate and regulating body temperature. The heart of the camel plays a crucial role in this adaptation, with specific genes expressed in cardiac tissue that are essential for mammalian adaptation, regulating cardiac function and responding to environmental stressors. One such gene, nebulin‐related‐anchoring protein (*NRAP*), is involved in the assembly of myofibrils and the transmission of force within the heart. In our study of the *NRAP* gene across various livestock species, including three camel species, we identified a camel‐specific exon region in the *NRAP* transcripts. This additional exon (exon 4) contains an open reading frame predicted in camels. To investigate its function, we generated knock‐in mice expressing camel *NRAP* exon 4. These ‘camelized mice’ exhibited normal phenotypic characteristics compared with wild‐type mice but showed elevated body temperatures under cold stress. Transcriptome analyses of the hearts from camelized mice under cold stress revealed differentially expressed inflammatory cytokine genes, known to influence cardiac function by modulating the contractility of cardiac muscle cells. We propose further investigations utilizing these camelized mice to explore these findings in greater depth.

## INTRODUCTION

Camels are among the mammals that have adapted to rapid temperature fluctuations, thriving in harsh environments such as deserts. Numerous physiological, behavioral and genetic studies have investigated the mechanisms behind camels’ environmental adaptations (Kandeel et al., 2022; Wu et al., 2014). However, the exact physiological pathways linking camel genomic components to their ability to withstand extreme environments remain unclear.

*NRAP* (nebulin‐related anchoring protein) is a member of the nebulin family and a highly evolutionarily conserved actin‐binding cytoskeletal protein that plays a role in myofibrillar assembly and force transmission in the heart (Luo et al., 1997; Truszkowska et al., 2017). Several studies on gene expression and population genomics have shown that *NRAP* is linked to environmental changes in habitat, including hibernation and hypoxia (Alderman et al., 2019; Capraro et al., 2019; Gagnaire et al., 2012). Genomic analyses of Yakut cattle, the world's northernmost breed, also revealed a Yakut‐specific missense mutation (H100Q) in *NRAP*, associated with cold adaptation, hibernation or torpor (Buggiotti et al., 2021). Additionally, a recent GWAS study identified *NRAP* variants associated with environmental adaptations in US Simmental cattle (Rowan et al., 2021).

These findings suggest that *NRAP* may play a significant functional role in environmental adaptation. Based on these findings, we investigated variations in the *NRAP* gene across various animals and discovered 26 amino acids (78‐mer, exon 4) that are uniquely present in camels. Given the expression characteristics of *NRAP* and the camel's known capacity for environmental adaptation, this particular exon 4 of camel *NRAP* could be implicated in providing a unique function that assists survival in extreme environmental conditions such as intense heat and cold. To study the role of camel *NRAP* exon 4 in extreme environments, we generated knock‐in mice (referred to as ‘camelized mice’) carrying the camel *NRAP* exon 4 using CRISPR/Cas9.

The ultimate goal of this study is to uncover the secrets of camels’ environmental adaptation using a camelized mouse model in which camel *NRAP* exon 4 is expressed. By observing temperature changes in camelized mice exposed to cold and performing RNA‐sequencing (RNA‐seq) transcriptomic profiling of the heart – a central component of the circulatory system where *NRAP* is highly expressed – we aim to establish a crucial foundation for understanding how camel exon 4 contributes to cold adaptation. This research will also pave the way for future analyses of heat adaptation mechanisms in camels. Ultimately, understanding the functional role of *NRAP* exon 4 in camels could lead to significant advancements in livestock management under extreme climate conditions, with broad implications for environmental adaptation studies in veterinary science and animal resilience strategies.

## MATERIALS AND METHODS

### Ethical approval

Mouse experiments were conducted in accordance with the guidelines of the Korean Food and Drug Administration at the GEM division of Macrogen Inc. (Seoul, Republic of Korea). The protocols underwent review and approval by the Institutional Animal Care and Use Committees (IACUC; mouse generation, MS‐2021‐01; cold exposure, CBNUA‐2138‐23‐02). All experiments were carried out in adherence to applicable legal guidelines.

### *NRAP* sequence analysis

A comparative analysis of *NRAP* amino acid sequences across 115 mammalian species, including camels, was performed using NCBI blast, multiple sequence alignment algorithms, and weblogo (https://weblogo.berkeley.edu/logo.cgi). The exon and intron boundaries of the *NRAP* transcript were analyzed for 14 mammalian species: camel (*Camelus ferus*), alpaca (*Vicugna pacos*), cattle (*Bos taurus*), zebu cattle (*Bos indicus*), bison (*Bison bison*), wild yak (*Bos mutus*), water buffalo (*Bubalus bubalis*), goat (*Capra hircus*), sheep (*Ovis aries*), pig (*Sus scrofa*), horse (*Equus caballus*), chicken (*Gallus gallus*), mouse (*Mus musculus*) and human (*Homo sapiens*). The boundaries were visualized using graphic maker (http://wormweb.org/exonintron), with a focus on the camel‐specific exon 4 and preserved splicing sites.

### Mouse housing

C57BL/6N and ICR mice, purchased from Orientbio Inc. (Seongnam, Republic of Korea), were housed at the GEM center of Macrogen Inc. under specific pathogen‐free conditions. The breeding room was maintained at a temperature of 22 ± 1°C and 50% humidity, with a 12 h light/dark cycle. All feed and individually ventilated cages were sterilized, and air conditioning units were equipped with filters to ensure a controlled environment. Carbon dioxide gas was used for euthanasia in accordance with standard protocols.

### Knock‐in strategy and sgRNA preparation

**FIGURE 1:**
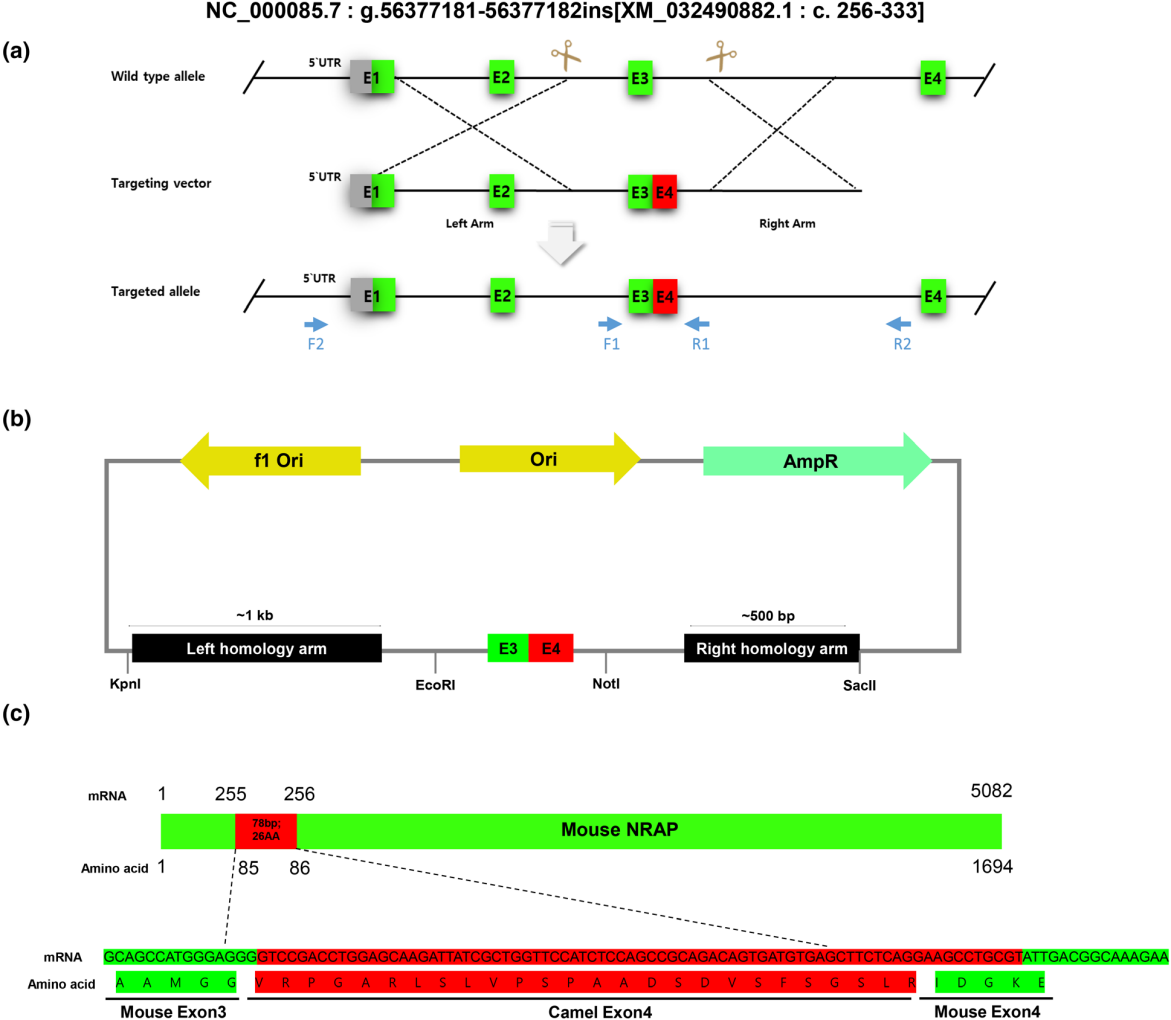
Overall designing for generation of *Nrap* ^*c.255ins78*^ mice. (a) Targeting strategy for knock‐in (KI). F1, 2 and R1, 2 indicate genotyping primer sites. The green box means mouse exons and the red box means camel exon 4. (b) Construction of the donor vector. To linearize the vector, KpnI and SacII restriction enzymes were positioned at both ends of the homology arms. Additionally, to facilitate genotyping using restriction fragment length polymorphism and prevent sgRNA off‐target effects, EcoRI and NotI restriction enzymes were placed in mouse introns 2 and 3. (c) Predicted coding sequence of camelized mice. The goal of our mouse model is to co‐express the mouse *Nrap* gene with camel exon 4 (26 amino acids). Thus, the length of the *Nrap* coding sequence of the *Nrap* ^*c.255ins78*^ mouse model is expected to be 5160 bp for mRNA and 1720 amino acids on an amino acid basis (based on NM_198059.4).

The knock‐in (KI) strategy was designed to insert camel *NRAP* exon 4 at the 3′ end of mouse *NRAP* exon 3 (NC_000085.7: g.56377181‐56377182ins (XM_032490882.1: c. 256‐333); abbreviated as *Nrap* ^*c.255ins78*^; Figure 1a). The insertion replaced approximately 500 bp of the genomic region, including mouse exon 3 and the adjacent intron, to enable natural splicing.

For sgRNA selection and off‐target analysis, we used the crispr design tool (https://chopchop.cbu.uib.no/), which systematically evaluates potential mismatch effects. The GRCm39/mm39 nucleotide sequence from the NCBI database (NC_000085.7, NM_198059) was referenced to determine the FASTA sequence for sgRNA targeting in intron 2 and intron 3, avoiding splicing sites. The selected crRNAs are listed in Table S1.

Double‐stranded DNA for *in vitro* transcription was constructed by PCR, with a T7 promoter sequence added to the 5′ end and a tracrRNA sequence to the 3′ end of the crRNA. *In vitro* transcription was performed by incubating 1 μg of the double‐stranded DNA template in 10 μl of 5× transcription buffer, 10 μl of rNTP (each 10 mm), 1.25 μl of RNase inhibitor (Invitrogen, Waltham, MA, USA) and 30 U of T7 RNA polymerase (EP0111; Thermo Fisher Scientific, USA) at 37°C for 2 h. After incubation, the reaction was treated with 2 U of DNase I (EO0381; Thermo Fisher Scientific, Waltham, MA, USA). The transcribed RNA was purified using the GeneArt™ RNA Purification Kit (A29377; Thermo Fisher Scientific) and verified via 2% agarose gel electrophoresis.

### Targeting vector construction

A targeting vector was designed to facilitate homology‐directed repair in mouse chromosomes, incorporating a mouse–camel hybrid exon composed of 88 bp of mouse exon 3 and 78 bp of camel exon 4. This hybrid exon was flanked by homology arms of approximately 500 bp to 1 kb. The left homology arm included part of exon 1 and exon 2 of the mouse gene, with the homology arm placements determined by the Cas9 cleavage sites identified by two selected crRNAs.

Six single‐stranded oligodeoxyribonucleotides (ssODNs) were synthesized with overlapping sense and antisense strands for PCR amplification. These ssODNs contained artificial mutations to avoid crRNA recognition and introduced EcoRI and NotI restriction sites for restriction fragment length polymorphism analysis. PCR amplification of the ssODNs was carried out using Q5 high‐fidelity polymerase (M0491L, NEB, Ipswich, MA, USA) under specific cycling conditions without a separate denaturation step. The resulting PCR products were purified.

Both the amplified ssODNs and the backbone vector, pBluescript SK(−) (Addgene, Cambridge, MA, USA) were digested with EcoRI and NotI (NEB) and ligated to construct the vector containing the hybrid exon structure.

The homology arms were amplified from mouse genomic DNA using Q5 high‐fidelity polymerase and specific restriction sites (SacII and KpnI; NEB). The amplified homology arm products and the vector containing the hybrid exon were digested with SacII and KpnI, followed by ligation to construct the final vector. The structure of the final vector is shown in Figure 1b, and the predicted coding sequence is shown in Figure 1c. The sequences of the ssODNs and primers used are listed in Table S2.

### Embryo collection and microinjection

For superovulation, 5‐ to 8‐week‐old C57BL/6N female mice were administered 7.5 IU of pregnant mare serum gonadotropin (Dsmbio, Uiwang, Republic of Korea), followed by 7.5 IU of human chorionic gonadotropin (hCG; Dsmbio) 48 h later via intraperitoneal injection. After hCG administration, the females were paired with C57BL/6N stud males for mating. Zygotes were collected from the oviducts 20 h post‐mating, after confirming the presence of a vaginal plug. The harvested zygotes were washed with Quinn's Advantage™ Medium with HEPES (ART‐1024; Cooper Surgical, Trumbull, CT, USA) and then transferred to KSOM medium (MR‐121‐D; Sigma‐Aldrich, St Louis and Burlington, MA, USA) in a CO_2_ incubator at 37°C. Microinjections were performed 24 h after hCG treatment.

The microinjection mixture contained Cas9 protein (CP1812071; Macrogen), purified sgRNAs, linearized donor DNA and SCR7 inhibitor (SML1546; Sigma‐Aldrich) and was injected into the pronucleus of 1‐cell zygotes in Quinn's Advantage™ Medium with HEPES at concentrations of 20/25/20 ng/μl and 1 μm, respectively. To enhance homology‐directed repair and inhibit the non‐homologous end‐joining pathway, SCR7 inhibitor (Hu et al., 2018) was included in the mixture. Following microinjection, embryos were incubated at 37°C for 2 h. The injected one‐cell embryos were surgically transplanted into the oviducts of pseudopregnant recipient ICR mice.

### Genotyping

Genomic DNA was extracted from mouse tails using the Axen™ Total DNA Mini Kit (MG‐P005‐50; Macrogen), following the manufacturer's instructions. PCR was performed using Q5® High‐Fidelity DNA Polymerase in a 40 μl reaction volume containing 8 μl of 5× Q5 Reaction Buffer, 8 μl of 5× Q5 High GC Enhancer, 0.8 μl of 10 mm dNTP mixture, 2 μl of 10 μm forward primer, 2 μl of 10 μm reverse primer, 200 ng of extracted genomic DNA and PCR‐grade water to complete the volume. The PCR conditions were as follows: initial denaturation at 98°C for 3 min; 35 cycles of 98°C for 10 s, 60°C for 15 s and 72°C for 30 s to 1 min; followed by a final extension at 72°C for 5 min.

The PCR products amplified using F1 and R1 primers were analyzed on a 2% agarose gel. To verify the correct insertion of donor DNA, the external region of the donor sequence (EOD) was amplified using F2 and R2 primers. Restriction fragment length polymorphism analysis was conducted on the amplified EOD using EcoRI and NotI restriction enzymes. The digested PCR products were separated on a 1% agarose gel.

Heterozygous alleles were further confirmed through PCR cloning (E1203S, NEB) followed by Sanger sequencing (Macrogen). The sequencing results were analyzed using NCBI's blast global alignment tool, and translation analysis was conducted with the expasy translate tool (https://web.expasy.org/translate/). The primer sequences used for genotyping are listed in Table S3.

### RT‐PCR

Total RNA was extracted from six organs (brain, lung, heart, liver, small intestine and kidney) of *Nrap* ^*c.255ins78*^ mice using the RNeasy Mini Kit (74104; Qiagen, Hilden, Germany) according to the manufacturer's instructions. The mice were euthanized using CO₂ gas. RNA quantity and purity were assessed with a Nanodrop spectrophotometer (Thermo Fisher Scientific). Complementary DNA (cDNA) was synthesized from 1 μg of total RNA from each tissue using the QuantiTect Reverse Transcription Kit (Qiagen).

RT‐PCR was conducted using Q5 High‐Fidelity DNA Polymerase. The 20 μl reaction mixture contained 4 μl of 5× Q5 Reaction Buffer, 4 μl of 5× Q5 High GC Enhancer, 0.4 μl of 10 mm dNTP mixture, 1 μl each of 10 μm forward and reverse primers, 1 μl of cDNA (diluted 1:10), and PCR‐grade water to make up the final volume. The PCR cycling conditions were: initial denaturation at 98°C for 3 min, followed by 35 cycles of 98°C for 10 s, 60°C for 15 s and 72°C for 30 s, with a final extension at 72°C for 5 min. Amplicons were verified by electrophoresis on 2% agarose gels, and sequencing was conducted via the Sanger method. The primer sequences used for RT‐PCR are listed in Table S3.

### Cold exposure

Cold exposure (CE) experiments were conducted to simulate extreme environmental conditions similar to a camel's natural habitat. The experiment was performed at 4°C for 12 h during the dark phase, following protocols established by Hu et al. (2022). Four 12‐week‐old *Nrap* ^*c.255ins78*^ homozygous KI male mice and four wild‐type (WT) male mice (controls) were exposed to the cold environment. Feed and water were available *ad libitum* throughout the experiment.

### Thermal imaging analysis and body weight measurements

Body temperatures were measured before and after CE using a handheld thermal imaging camera (HT‐18; Hti‐Xintai, China), with a resolution of 220 × 160 pixels, an accuracy of ±2°C and a sensitivity of 0.08°C. Thermal images were processed and analyzed using irimagetools software (Vogel et al., 2016).

Mouse body weight and tissue samples were measured using a precision scale (BP4100S; Sartorius, Göttingen, Germany), with a sensitivity of 0.01 g and a maximum capacity of 4.1 kg.

### RNA‐seq

Twelve‐week‐old male *Nrap* ^*c.255ins78*^ KI homozygous mice (*n* = 4) and WT controls (*n* = 4) were sacrificed following cold exposure, with euthanasia performed via CO_2_, in accordance with IACUC guidelines. Heart tissues were harvested and homogenized using a homogenizer (TH‐02; Qiagen). Total RNA was extracted using the RNeasy Mini Kit according to the manufacturer's protocol. The quantity and purity of the extracted RNA were evaluated using TapeStation RNA ScreenTape (Agilent Technologies, Santa Clara, CA, USA).

A cDNA library was constructed from the total RNA using the TruSeq Stranded mRNA Prep Kit (Illumina, San Diego, CA, USA), following the guidelines provided in the TruSeq Stranded mRNA Reference Guide no. 1000000040498 v00. The quality of the constructed libraries was assessed using TapeStation D1000 ScreenTape (Agilent Technologies). Sequencing was performed on an Illumina NovaSeq 6000 system (Illumina) with paired‐end reads.

### Differentially expressed gene and functional enrichment analysis

The quality of raw read data for each sample was assessed using fastqc software v0.11.7. Adaptor trimming was performed on the raw read data using trimmomatic v0.38. The trimmed data were then aligned to the reference genome (GRCm39, GCF_000001635.27) obtained from the Ensembl genome browser using hisat2 v2.1.0. Raw counts of mapped sequence reads were calculated based on the exons in the *Mus musculus* GTF v111 (Ensembl) using featureCounts from the subread package v1.6.3.

Differential expression analyses were conducted using edger v3.26.5, with raw counts normalized using the TMM (trimmed mean of *M*‐values) method. The criteria for identifying differentially expressed genes (DEGs) in the heart between *Nrap* ^*c.255ins78*^ and WT groups under cold exposure conditions included a significance threshold of *p* < 0.01 and an absolute log_2_ fold change of ≥1.

The identified DEGs were annotated to Gene Ontology (GO) terms and Kyoto Encyclopedia of Genes and Genomes (KEGG) pathways using david v6.8. Enrichment analyses for GO biological process (BP) terms and KEGG pathways were performed with thresholds of *p* < 0.01 and counts ≥3. Enriched GO terms were clustered based on similarity and visualized using treemaps generated by revigo (Supek et al., 2011). The GO functional enrichment analyses were conducted using the same threshold criteria and represented by –log_10_ *p* and fold enrichment.

### RT‐qPCR validation

Multiple quantitative PCRs (qPCRs) were performed using modified oligonucleotides in 96‐well plates (HSP9601; Bio‐Rad, Hercules, CA, USA) to validate the results obtained from RNA‐seq. To achieve appropriate fluorescence detection, the 5′ end of the probes was modified with FAM, HEX and Cy5 fluorescent dyes, while the 3′ end was modified with BHQ2 and TAMRA quenchers. The probes were designed to have an annealing temperature at least 5°C higher than that of the amplifying primer pair and were limited to a maximum length of 35 bp.

*In silico* analysis performed using the UCSC Genome Browser was utilized to minimize the occurrence of hairpins and primer dimers. The qPCR reactions were conducted using the QuantiNova Multiplex Kit (Qiagen) and TaqMan™ Universal PCR Master Mix (Thermo Fisher Scientific), with *β*‐actin expression used as a control. Relative quantification of mRNA expression was calculated using the $2^{-∆∆C_{t}}$ method after normalization to the *β‐actin* gene, and results were presented as the average relative fold change. The sequences of the primers and probes used in the qPCR are provided in Table S4.

### Protein structure prediction

To assess the preservation of functional motifs and alterations in the three‐dimensional structure of the protein, domain prediction was conducted using the SMART (Schultz et al., 2000) and InterPro (Apweiler et al., 2000) databases. Protein modeling was carried out utilizing swiss‐model (Waterhouse et al., 2018). The predicted protein structure was visualized using chimerax v1.8, employing the template amino acid sequences NP_932307.2 (Mouse) and XP_032346773.1 (Camel).

### Statistics

All data are presented as mean ± SEM. RNA‐seq data analysis and visualization were performed using r v.4.3.3 statistical software. The qPCR experiments were independently repeated three times. Statistical significance was determined using a paired *t*‐test, with *p*‐values less than 0.05 considered statistically significant.

## RESULTS

### Detection of camelid‐specific exon in *NRAP* transcripts

Analysis of *NRAP* sequences across 115 mammalian species revealed a unique 26 amino acid insertion (78mer, Exon 4) in camels (Figure S1). This sequence was specifically expressed in the *NRAP* gene of two camel species, *Camelus ferus* and *Camelus dromedarius*, as well as in a closely related species, the alpaca (*Vicugna pacos*). Further analysis using NCBI blast confirmed that this 26‐amino‐acid sequence is not conserved in other species, although traces of the corresponding DNA sequence were detected in five whale species (*Stenella coeruleoalba*, *Delphinus delphis*, *Balaenoptera acutorostrata*, *Lagenorhynchus albirostris* and *Orcinus orca*) (Table S5). The boundaries of this exon‐intron region show that the camel‐specific exon 4 includes conserved splicing sites (Figure S2).

Interestingly, sequence analysis of homologous regions in other closely related species revealed that, except for chickens, pigs and horses, all species possessed homologous DNA sequences between exon 3 and exon 4, with species‐specific amino acid substitutions in this region (Table S6). Despite these substitutions, the region maintained an open reading frame in its genes. Notably, only camels and alpacas appear to utilize this region to express the *NRAP* gene.

### Generation of *Nrap* ^*c.255ins78*^ mice

**FIGURE 2:**
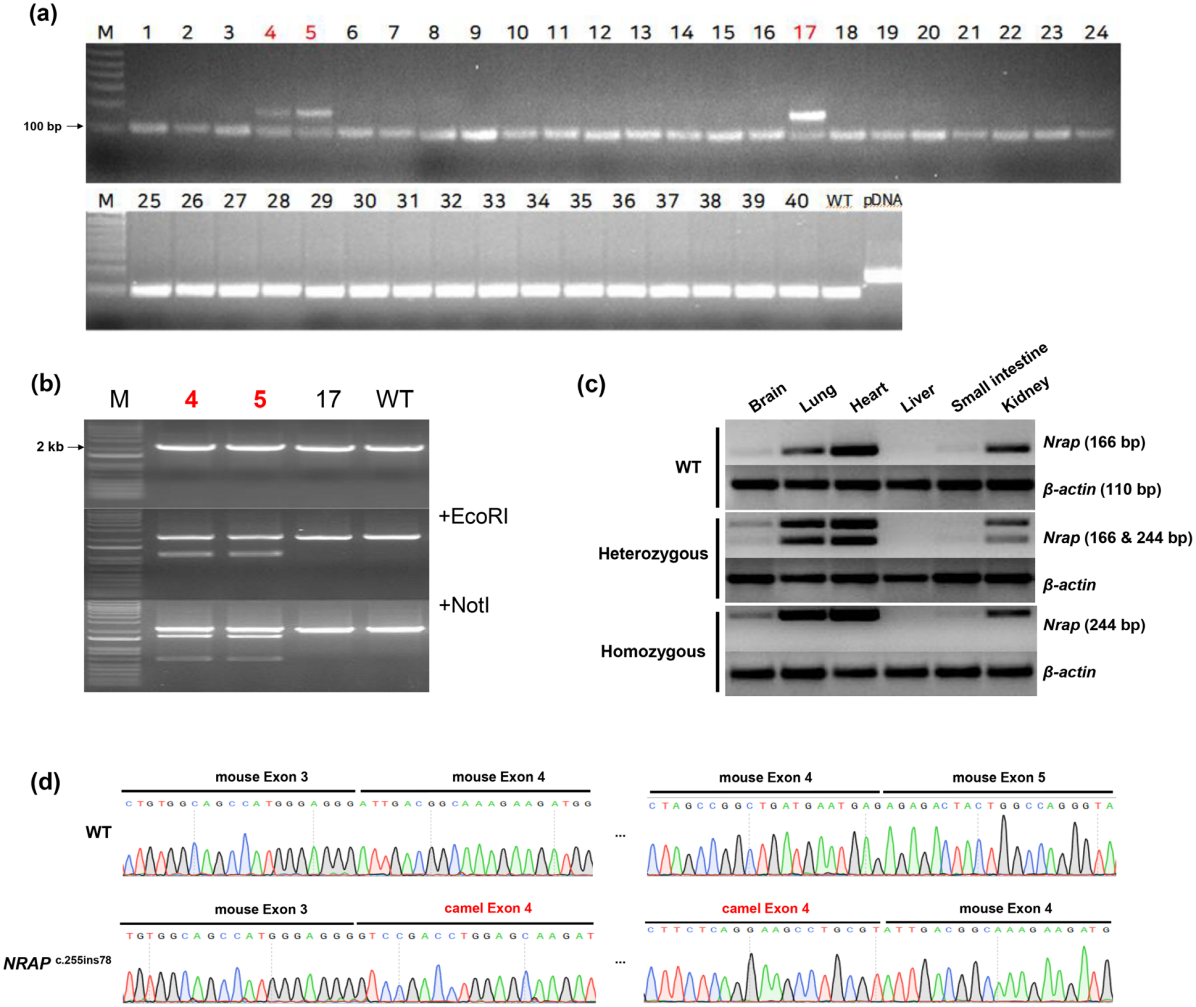
Generation of *Nrap* ^*c.255ins78*^ mice. (a) Genotyping of founder pups via PCR. To identify the insertion of the 78 bp camel exon 4 sequence, the mouse exon 3 region was amplified. The resulting amplicon produced a 95 bp band for wild‐type (WT) and a 173 bp band for plasmid DNA and positive candidate samples. (b) Restriction fragment length polymorphism analysis of knock‐in (KI) candidates. For the external region of donor (EOD) PCR, KI types were differentiated using artificially inserted restriction enzyme sites. To confirm on‐target insertion, the EOD PCR product was treated with EcoRI and NotI. For positive KI confirmation, treatment with EcoRI resulted in bands at 1073 and 1162 bp, respectively, whereas NotI treatment yielded bands at 1601 and 634 bp. (c) A comparison of the mouse *NRAP* mRNA expression level between cardiac muscle (heart) and non‐muscle tissue (brain, lung, liver, small intestine and kidney) through RT‐PCR. We observed the fused mRNA of mouse exon 3 and camel exon 4 in not only the heart but also the brain, lung, small intestine and kidney. However, it showed weak expression in the brain and small intestine, and no expression was confirmed in the liver. The experiments on WT, homozygous and heterozygous mice were conducted at the age of 12 weeks old, which is adult. (d) Sanger sequencing by RT‐PCR product. We confirmed by sequencing that the *Nrap* gene in *Nrap* ^*c.255ins78*^ mice was normally spliced and transcribed.

A total of 86 fertilized embryos were transplanted into four ICR surrogate mice, all of which successfully carried to term. Nineteen days later, 40 offspring were born (Table S7). To confirm the insertion of the camel exon, PCR assays were performed on the offspring, identifying three candidate mice (Figure 2a). To rule out non‐targeted insertions, primers (F2 and R2) were designed outside the donor DNA insertion site, and alleles were further validated using restriction fragment length polymorphism analysis (Figure 2b). The PCR‐cloned alleles were sequenced via Sanger sequencing (Figure S3a), confirming two positive founder mice, *Nrap* ^*c.255ins78*^. The genetically verified *Nrap* ^*c.255ins78*^ mice exhibited no notable phenotypic abnormalities in terms of physical appearance, including body and heart morphology (Figure S3b). To establish a stable line, natural mating was conducted, advancing the lineage to the F5 generation, with several homozygous mice successfully obtained (Figure S3c).

### Expression of hybrid *Nrap* gene

To confirm the correct expression of the inserted camel exon, we performed RT‐PCR, enabling verification of expression through size‐based separation of amplified products. All mice used in this experiment were 12 weeks old, including homozygous mice from six generations, heterozygous mice from five generations and WT controls. Expression was evaluated across the heart (a muscle tissue), as well as in the brain, lung, liver, small intestine and kidney (non‐muscle tissues) for each group. The transcript was detected in the heart, lung and kidney of both heterozygous and homozygous mice (Figure 2c). Sanger sequencing further confirmed that the mRNA of the fusion structure, including mouse exon 3, camel exon 4 and mouse exons 4 and 5, was successfully transcribed (Figure 2d).

### Increase in body temperature of *Nrap* ^*c.255ins78*^ Mice during cold exposure

**FIGURE 3:**
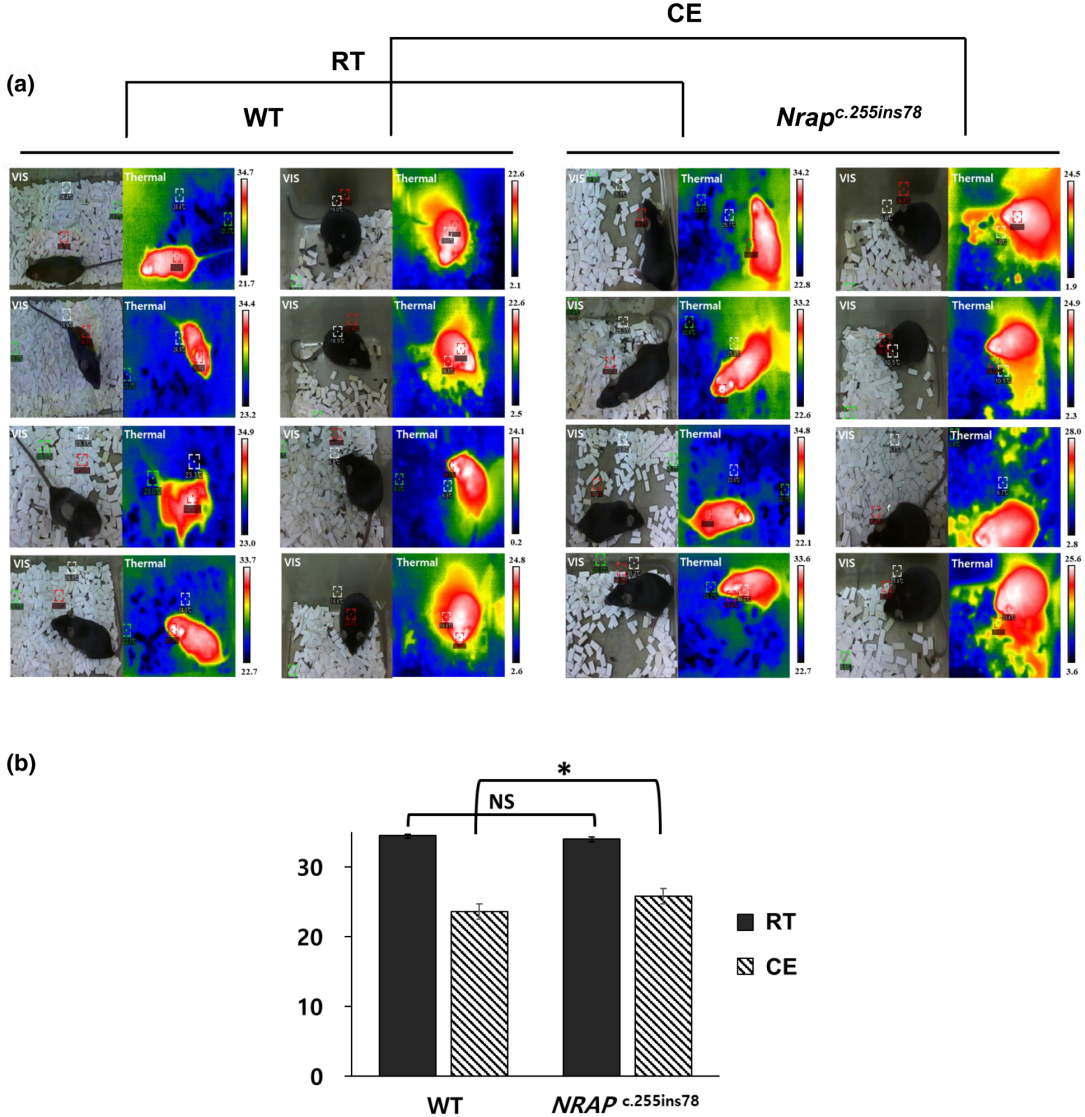
Body temperature measurement using a thermal imaging camera: visual (VIS) and thermal images (Thermal). (a) Mouse body temperature measurement at room temperature (RT) and under cold exposure (CE) using a thermal imaging camera. (b) Results of body temperature in RT and CE. The values represent the mean ± SEM (*n* = 4 per group). *Significantly different from the wild‐type (WT) group at *p* < 0.05.

**FIGURE 4:**
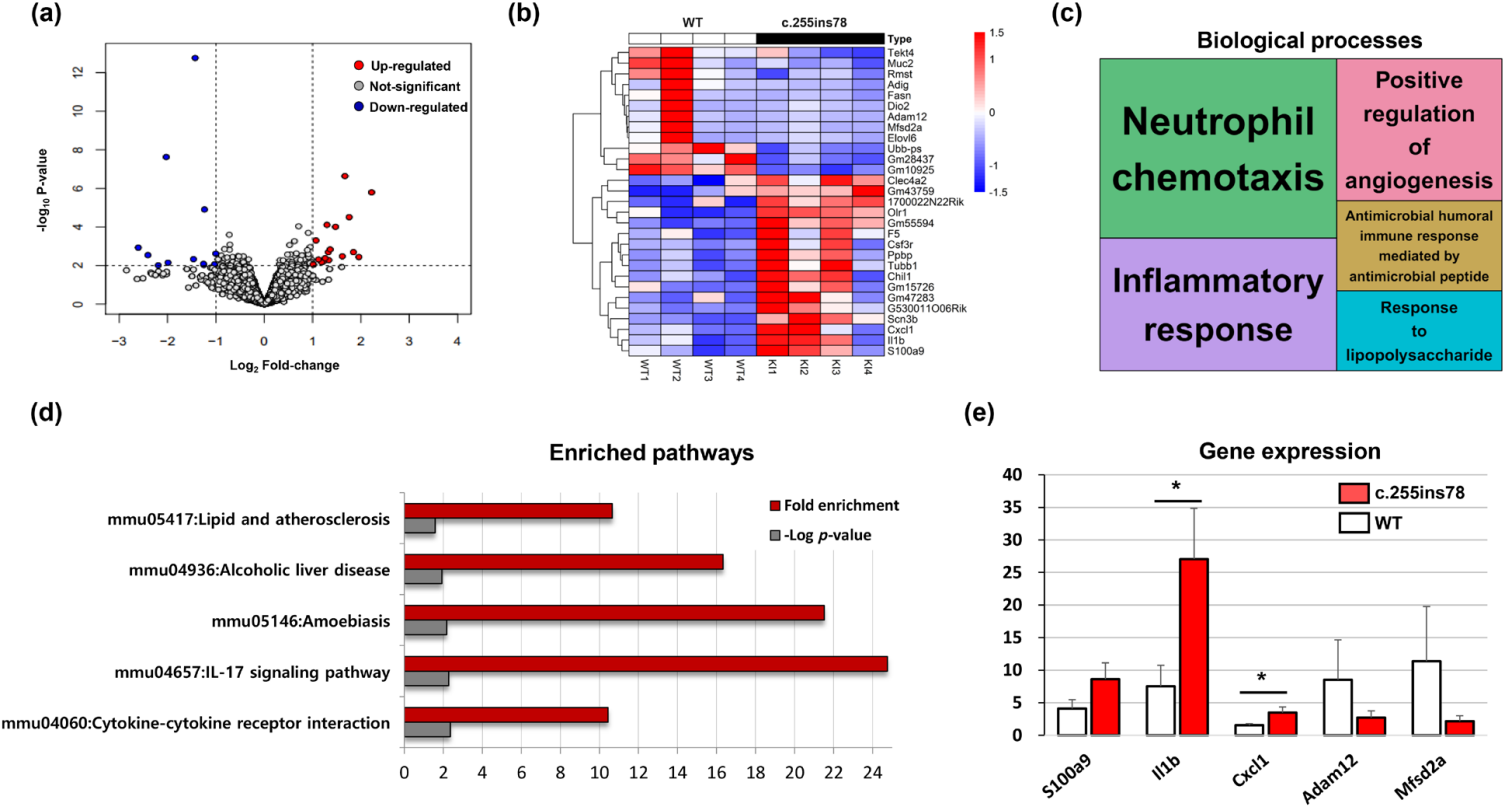
Transcriptome in the heart under cold exposure. (a) Volcano plots of RNA changes between the *Nrap* ^*c.255ins78*^ mice and the wild‐type (WT) mice after cold exposure. The *x*‐ and *y*‐axes of the volcano plots represent the log_2_ fold‐change and –log_10_ *p*, respectively. (b) Heatmap of differentially expressed genes (DEGs) shows the expression patterns in the heart. (c) Enrichment treemap of Gene Ontology biological processes in the heart tissue with –log_10_ *p* > 1. (d) The Kyoto Encyclopedia of Genes and Genomes (KEGG) enriched pathways are represented. (e) It was confirmed that the qPCR results of five DEGs in the heart tissue were similar to the RNA‐sequencing result. Comparisons between two groups and individuals were performed and evaluated for statistical significance. *p < 0.05.

After 12 h of CE at 4°C during the dark phase, body temperature was immediately measured using a thermal imaging camera. The experiment involved eight mice (*n* = 4 per group), with measurements taken both before (room temperature) and after CE (Figure 3a). Notably, during the CE, the change in body temperature in KI mice was significantly less than that of WT mice (*p* < 0.05; Figure 4b). In contrast, no significant changes in body weight were observed after CE (Table S8).

### Heart transcriptomes under cold exposure

A total of 304 million raw reads were generated from eight samples, with an average of 38 million reads per sample (Table S9). Differential expression analysis comparing gene activity in heart tissue under CE conditions was visualized using volcano plots (Figure 4a) and a heatmap (Figure 4b). A total of 29 DEGs were identified, of which 12 genes were downregulated, comprising *Adam12*, *Mfsd2a*, *Dio2*, *Gm28437*, *Elovl6*, *Adig*, *Gm10925*, *Tekt4*, *Muc2*, *Ubb‐ps*, *Rmst* and *Fasn*. Meanwhile, 17 genes were upregulated, comprising *Clec4a2*, *Gm43759*, *Scn3b*, *1700022N22Rik*, *Csf3r*, *Chil1*, *Cxcl1*, *Olr1*, *F5*, *Il1b*, *Ppbp*, *Gm47283*, *Gm55594*, *Tubb1*, *S100a9*, *Gm15726* and *G530011O06Rik* (Table S10).

Functional enrichment analysis using GO BP and KEGG pathways revealed significant enrichment for these DEGs in heart tissue. Notably, GO BP terms such as ‘neutrophil chemotaxis’ (GO:0030593, *p* = 1.15 × 10^−6^) and ‘inflammatory response’ (GO:0006954, *p* = 4.01 × 10^−5^) showed significant involvement (Figure 4c). KEGG pathway analysis further highlighted key pathways, including ‘Cytokine–cytokine receptor interaction’ (mmu04060, *p* = 0.004), ‘IL‐17 signaling pathway’ (mmu04657, *p* = 0.005), ‘Amoebiasis’ (mmu05146, *p* = 0.007), ‘Alcoholic liver disease’ (mmu04936, *p* = 0.012) and ‘Lipid and atherosclerosis’ (mmu05417, *p* = 0.026) (Table S11, Figure 4d).

To confirm the technical reliability of the RNA‐seq data, we randomly selected five genes (*S100a9*, *Il1b*, *Cxcl1*, *Adam12* and *Mfsd2a*) from the DEGs for validation through qPCR. The qPCR results closely matched the transcriptome analysis findings (Figure 4e). Across the tested groups (*n* = 4), the mean expression levels of these genes were generally consistent with the RNA‐seq data. Notably, *Il1b* (*p* = 0.032) and *Cxcl1* (*p* = 0.028) showed statistically significant differences between the two groups.

### Protein prediction

Domain and protein structure analysis of *NRAP* revealed that the WT *Nrap* protein contains 41 nebulin domains, whereas the *Nrap* ^c.255ins78^ protein is predicted to have 40 domains (Figure S4a). The three‐dimensional structural analysis showed that the 26 inserted camel amino acids were seamlessly integrated between the existing domains in both the mouse and camel proteins, with no significant alterations to the overall protein structure. This suggests that the structural stability of the protein remains unaffected (Figure S4b).

## DISCUSSION

In this study, we identified a unique exon 4 in the *NRAP* gene specific to camels. Building on this discovery, we employed the CRISPR/Cas9 system to create a ‘camelized’ mouse model by integrating the predicted exon 4 from the camel *NRAP* gene into the mouse genome. This method enabled us to engineer a mouse with camel‐like genetic traits while maintaining controlled gene expression through the endogenous mouse promoter and splicing sites, thus reducing interspecies expression differences. However, the potential for mosaicism, where different cells within the same organism carry distinct genetic profiles, remains a known challenge in CRISPR/Cas9‐engineered models (Mehravar et al., 2019; Taylor et al., 2014). Additionally, CRISPR/Cas9 may have caused off‐target effects in the mouse genome, potentially obscuring the full impact of the intended mutation. To mitigate this risk, we crossbred the edited mice with WT mice up to the F4 generation, followed by inbreeding to establish homozygous lines.

Our findings demonstrated that the camelized mice exhibited improved adaptation to cold environments compared to WT mice, as evidenced by significant differences in body temperature (*p* < 0.05). Although difficult to quantify, there was also a visually observable increase in activity levels after cold exposure. The underlying mechanisms for these temperature changes may include cardiovascular regulation, mitochondrial function, and activation of brown adipose tissue.

To investigate the role of exon 4 of the *NRAP* gene expressed in the heart, RNA‐seq was performed on cardiac tissue. The heart is a vital circulatory organ, not only responsible for distributing essential substances like blood and hormones to other organs and regulating heart rate but also playing a critical role in temperature regulation (González‐Alonso, 2012). Structurally, it comprises two atria and two ventricles, which facilitate both systemic and pulmonary circulation through arterial and venous blood flow (Li et al., 2009; Polak‐Iwaniuk et al., 2019). When exposed to low temperatures, mammalian hearts are influenced by various factors, such as increased blood pressure within the cardiovascular system (Houdas et al., 1992; Meyer et al., 2010). In addition, inflammatory cytokines contribute to changes in intracellular calcium transport and myocardial contraction, which can affect blood pressure regulation and the development of hypertension, particularly with regard to kidney function (Ramadan et al., 2011; Wen & Crowley, 2018). Understanding how exon 4 of *NRAP* contributes to these processes could provide valuable insights into cardiac adaptation mechanisms in cold environments.

In our investigation, we identified significant involvement of the ‘Cytokine–cytokine receptor interaction’ and ‘IL‐17 signaling’ pathways in the secretion of inflammatory cytokines, as demonstrated by KEGG pathway analysis. The upregulation of *Il1b* was particularly intriguing, as it plays a pivotal role as a mediator of inflammatory cytokines, influencing processes such as cell proliferation, differentiation and apoptosis (Dinarello, 2010, 2011). This cytokine is notably implicated in inflammatory hypersensitivity, especially through its induction of cyclooxygenase‐2 in the central nervous system.

Another key upregulated gene, *Cxcl1*, a member of the CXC chemokine family, acts as a chemoattractant for neutrophils and other immune cells at sites of tissue damage, thus playing a crucial role in regulating immune and inflammatory responses (Amiri & Richmond, 2003; Jin et al., 2014). Additionally, we observed an interesting increase in *S100a9*, which is part of the S100 protein family and contains two EF‐hand calcium‐binding motifs. This cytoplasmic protein is primarily expressed by phagocytes and is often characterized as a cytokine‐like protein owing to its release from inflamed tissues (Gebhardt et al., 2006; Simard et al., 2013). The secretion of inflammatory cytokines induced by S100a9 has been associated with calcium depletion in vesicles (Clark et al., 2017).

While *S100a8*, another member of the S100 family, was not selected as a DEG owing to it not meeting the significance threshold (*p* < 0.01), it did exhibit increased expression at the *p* < 0.05 level. Similar to *S100a9*, *S100a8* is involved in regulating the cell cycle and differentiation, and it can inhibit casein kinase (Millward et al., 1998). Notably, intracellular *S100a9* has been shown to alter mitochondrial homeostasis in neutrophils (Li et al., 2019). Studies in mice deficient in *S100a9* demonstrated protection from systemic *Staphylococcus aureus* infection owing to a reduced bacterial burden in the heart, suggesting a potential organ‐specific function for *S100a9* (Monteith et al., 2021). Conversely, overexpression of *S100a9* in mice appears to increase inflammatory cytokines in the heart and stimulates the production of reactive oxygen species, leading to the secretion of additional cytokines and chemokines (Simard et al., 2013). Our analysis of heart transcription under cold exposure conditions provides experimental confirmation of these findings.

Although the *S100a9* gene did not yield statistically significant results in qPCR (*p* = 0.071), this discrepancy is probably attributable to variations in normalization and data processing methods between RNA‐seq and qPCR. Interestingly, the relative expression levels measured by qPCR across all eight samples closely mirrored the heatmap results derived from the RNA‐seq data (Figure 4b). Notably, the distinct expression profile of the WT2 sample was evident among the downregulated DEGs, as this sample displayed markedly higher expression levels. This unique expression pattern in the WT2 sample was also reflected in the qPCR results, potentially explaining the non‐significant *p*‐values for the *Adam12* and *Mfsd2a* genes in qPCR, given that their expression was strongly influenced by the outlier profile of the WT2 sample.

In conclusion, we successfully developed and stabilized the camelized mouse line, *Nrap* ^c.255ins78^, to investigate the function of camel exon 4 in relation to cold adaptation. Our findings indicate that *Nrap* ^c.255ins78^ exhibits a more cold‐tolerant phenotype compared with WT mice. Transcriptomic analysis revealed that the heart of *Nrap* ^c.255ins78^ emphasizes pathways related to neutrophil chemotaxis and the inflammatory response. Given that the heart is the most critical organ in the circulatory system, changes observed in this organ are expected to have significant implications for the overall circulatory system. Although this study was primarily focused on the heart, future research should explore the roles of other circulatory organs, such as the lungs and kidneys, as well as the contribution of brown adipose tissue to cold adaptation. While our transcriptome analysis is based on predictions and we did not use actual camel samples, which means that we cannot entirely rule out the possibility of a pseudoexon, our cold exposure experiments and transcriptomic analyses clearly demonstrate the expression of exon 4. Furthermore, incorporating cell‐based heat stress experiments may yield additional valuable insights. Ultimately, our research serves as an important initial investigation into the mechanisms of extreme environmental adaptation in camels, utilizing a camel‐specific exon motif.

## AUTHOR CONTRIBUTIONS

**Sung‐Yeon Lee:** Conceptualization; investigation; resources; software; validation; visualization; writing – original draft; writing – review and editing. **Bo‐Young Lee:** Conceptualization; data curation; formal analysis; investigation; software; visualization; writing – original draft. **Byeonghwi Lim:** Data curation; investigation; methodology; software. **Rasel Uzzaman:** Formal analysis; investigation; validation. **Goo Jang:** Methodology; software. **Kwan‐Suk Kim:** Conceptualization; funding acquisition; investigation; project administration; resources; supervision; writing – original draft; writing – review and editing.

## CONFLICT OF INTEREST STATEMENT

The authors declare no conflict of interest.

## Supporting information


Figure S1.
Figure S2.
Figure S3.
Figure S4.

Table S1.

## Data Availability

The raw transcriptome sequencing data has been deposited at NCBI under accession number PRJNA1112111. We conducted the analysis using the following protein accession numbers: *Camelus ferus* (XP_032346773.1), *Camelus bactrianus* (XP_010968128.1), *Camelus dromedarius* (XP_031317641.1), *Vicugna pacos* (XP_031538451.1), *Bos taurus* (XP_005225802.2), *Bos indicus* (XP_019844179.1), *Bison bison* (XP_010857615.1), *Bos mutus* (XP_005888717.1), *Bublus bubalis* (XP_045020061.1), *Capra hircus* (XP_017896966.1), *Ovis aries* (XP_042095042.1), *Gallus gallus* (XP_003641574.1), *Sus scrofa* (XP_020927848.1), *Equus caballus* (XP_005602278.2), *Homo sapiens* (NP_932326.2) and *Mus musculus* (NP_932307.2).

## References

[age13490-bib-0001] Alderman, S.L. , Crossley, D.A. , Elsey, R.M. & Gillis, T.E. (2019) Hypoxia‐induced reprogramming of the cardiac phenotype in American alligators (*Alligator mississippiensis*) revealed by quantitative proteomics. Scientific Reports, 9(1), 8592. 31197188 10.1038/s41598-019-45023-3 PMC6565670

[age13490-bib-0002] Amiri, K.I. & Richmond, A. (2003) Fine tuning the transcriptional regulation of the CXCL1 chemokine. Progress in Nucleic Acid Research and Molecular Biology, 74, 1–36. 14510072 10.1016/s0079-6603(03)01009-2 PMC3140403

[age13490-bib-0003] Apweiler, R. , Attwood, T.K. , Bairoch, A. , Bateman, A. , Birney, E. , Biswas, M. et al. (2000) InterPro – an integrated documentation resource for protein families, domains and functional sites. Bioinformatics, 16(12), 1145–1150. 11159333 10.1093/bioinformatics/16.12.1145

[age13490-bib-0004] Buggiotti, L. , Yurchenko, A.A. , Yudin, N.S. , Vander Jagt, C.J. , Vorobieva, N.V. , Kusliy, M.A. et al. (2021) Demographic history, adaptation, and NRAP convergent evolution at amino acid residue 100 in the world northernmost cattle from Siberia. Molecular Biology and Evolution, 38(8), 3093–3110. Available from: 10.1093/molbev/msab078 33784744 PMC8321547

[age13490-bib-0005] Capraro, A. , O'Meally, D. , Waters, S.A. , Patel, H.R. , Georges, A. & Waters, P.D. (2019) Waking the sleeping dragon: gene expression profiling reveals adaptive strategies of the hibernating reptile *Pogona vitticeps* . BMC Genomics, 20, 460. Available from: 10.1186/s12864-019-5750-x 31170930 PMC6555745

[age13490-bib-0007] Clark, A.L. , Kanekura, K. , Lavagnino, Z. , Spears, L.D. , Abreu, D. , Mahadevan, J. et al. (2017) Targeting cellular calcium homeostasis to prevent cytokine‐mediated beta cell death. Scientific Reports, 7(1), 5611. 28717166 10.1038/s41598-017-05935-4 PMC5514111

[age13490-bib-0008] Dinarello, C.A. (2010) IL‐1: discoveries, controversies and future directions. European Journal of Immunology, 40(3), 599–606. Available from: 10.1002/eji.201040319 20201008

[age13490-bib-0009] Dinarello, C.A. (2011) Interleukin‐1 in the pathogenesis and treatment of inflammatory diseases. Blood, 117(14), 3720–3732. 21304099 10.1182/blood-2010-07-273417 PMC3083294

[age13490-bib-0011] Gagnaire, P.A. , Normandeau, E. , Côté, C. , Hansen, M.M. & Bernatchez, L. (2012) The genetic consequences of spatially varying selection in the Panmictic American eel (*Anguilla rostrata*). Genetics, 190(2), 725–736. 22135355 10.1534/genetics.111.134825 PMC3276646

[age13490-bib-0012] Gebhardt, C. , Németh, J. , Angel, P. & Hess, J. (2006) S100A8 and S100A9 in inflammation and cancer. Biochemical Pharmacology, 72(11), 1622–1631. 16846592 10.1016/j.bcp.2006.05.017

[age13490-bib-0013] González‐Alonso, J. (2012) Human thermoregulation and the cardiovascular system. Experimental Physiology, 97(3), 340–346. Available from: 10.1113/expphysiol.2011.058701 22227198

[age13490-bib-0014] Houdas, Y. , Dekiunder, G. & Lecroart, J.‐L. (1992) Cold exposure and ischemic heart disease. International Journal of Sports Medicine, 13, 179–181. 10.1055/s-2007-1024632 1483767

[age13490-bib-0015] Hu, Y. , Liu, Y. & Li, S. (2022) Effect of acute cold stress on neuroethology in mice and establishment of its model. Animals, 12(19), 2671. 10.3390/ani12192671 PMC9559653 36230412

[age13490-bib-0016] Hu, Z. , Shi, Z. , Guo, X. , Jiang, B. , Wang, G. , Luo, D. et al. (2018) Ligase IV inhibitor SCR7 enhances gene editing directed by CRISPR‐Cas9 and ssODN in human cancer cells. Cell & Bioscience, 8(12), 12. 29468011 10.1186/s13578-018-0200-z PMC5819182

[age13490-bib-0017] Jin, L. , Batra, S. , Douda, D.N. , Palaniyar, N. & Jeyaseelan, S. (2014) CXCL1 contributes to host defense in polymicrobial sepsis via modulating T cell and neutrophil functions. The Journal of Immunology, 193(7), 3549–3558. 25172493 10.4049/jimmunol.1401138 PMC4170008

[age13490-bib-0018] Kandeel, M. , Al‐Taher, A. , Venugopala, K.N. , Marzok, M. , Morsy, M. & Nagaraja, S. (2022) Camel proteins and enzymes: a growing resource for functional evolution and environmental adaptation. Frontiers in Veterinary Science, 9, 911511. Available from: 10.3389/fvets.2022.911511 35903143 PMC9315206

[age13490-bib-0019] Li, W. , Xu, X. , Chen, B. , Zhang, J. , Ren, X. , Guo, H. et al. (2009) A novel heart structure and work mode hypothesis: parasitic ventricle theory. Medical Hypotheses, 72(5), 541–543. 19201102 10.1016/j.mehy.2008.10.028

[age13490-bib-0020] Li, Y. , Chen, B. , Yang, X. , Zhang, C. , Jiao, Y. , Li, P. et al. (2019) S100a8/a9 signaling causes mitochondrial dysfunction and cardiomyocyte death in response to ischemic/reperfusion injury. Circulation, 140(9), 751–764. 31220942 10.1161/CIRCULATIONAHA.118.039262

[age13490-bib-0022] Luo, G. , Zhang, J.Q. , Nguyen, T.‐P. , Herrera, A.H. , Paterson, B. & Horowits, R. (1997) Complete cDNA sequence and tissue localization of N‐RAP, a novel nebulin‐related protein of striated muscle. Cell Motility and the Cytoskeleton, 38, 75–90. 9295142 10.1002/(SICI)1097-0169(1997)38:1<75::AID-CM7>3.0.CO;2-G

[age13490-bib-0023] Mehravar, M. , Shirazi, A. , Nazari, M. & Banan, M. (2019) Mosaicism in CRISPR/Cas9‐mediated genome editing. Developmental Biology, 445(2), 156–162. 30359560 10.1016/j.ydbio.2018.10.008

[age13490-bib-0024] Meyer, P. , Guiraud Msc, T. , Curnier, D. , Juneau, M. , Gayda, M. , Nozza, A. et al. (2010) Exposure to extreme cold lowers the ischemic threshold in coronary artery disease patients. Canadian Journal of Cardiology, 26(2), e50–e53. 20151059 10.1016/s0828-282x(10)70007-6 PMC2851392

[age13490-bib-0025] Millward, T.A. , Heizmann, C.W. , Schä Fer, B.W. & Hemmings, B.A. (1998) Calcium regulation of Ndr protein kinase mediated by S100 calcium‐binding proteins. The EMBO Journal, 17(20), 5913–5922. 9774336 10.1093/emboj/17.20.5913 PMC1170919

[age13490-bib-0026] Monteith, A.J. , Miller, J.M. , Maxwell, C.N. , Chazin, W.J. & Skaar, E.P. (2021) S100A9 modulates neutrophil extracellular trap formation thereby enhancing macrophage killing of bacterial pathogens. The Journal of Immunology, 206(1_Supplement), 110.02.

[age13490-bib-0027] Polak‐Iwaniuk, A. , Harasim‐Symbor, E. , Gołaszewska, K. & Chabowski, A. (2019) How hypertension affects heart metabolism. Frontiers in Physiology, 10, 435. 31040794 10.3389/fphys.2019.00435 PMC6476990

[age13490-bib-0028] Ramadan, J.W. , Steiner, S.R. , O'Neill, C.M. & Nunemaker, C.S. (2011) The central role of calcium in the effects of cytokines on beta‐cell function: implications for type 1 and type 2 diabetes. Cell Calcium, 50(6), 481–490. 21944825 10.1016/j.ceca.2011.08.005 PMC3223281

[age13490-bib-0029] Rowan, T.N. , Durbin, H.J. , Seabury, C.M. , Schnabel, R.D. & Decker, J.E. (2021) Powerful detection of polygenic selection and evidence of environmental adaptation in US beef cattle. PLoS Genetics, 17(7), e1009652. 34292938 10.1371/journal.pgen.1009652 PMC8297814

[age13490-bib-0030] Schultz, J. , Copley, R.R. , Doerks, T. , Ponting, C.P. & Bork, P. (2000) SMART: a web‐based tool for the study of genetically mobile domains. Nucleic Acids Research, 28(1), 231–234. 10592234 10.1093/nar/28.1.231 PMC102444

[age13490-bib-0031] Simard, J.C. , Cesaro, A. , Chapeton‐Montes, J. , Tardif, M. , Antoine, F. , Girard, D. et al. (2013) S100A8 and S100A9 induce cytokine expression and regulate the NLRP3 Inflammasome via ROS‐dependent activation of NF‐kB1. PLoS One, 8(8), e72138. 23977231 10.1371/journal.pone.0072138 PMC3747084

[age13490-bib-0032] Supek, F. , Bošnjak, M. , Škunca, N. & Šmuc, T. (2011) Revigo summarizes and visualizes long lists of gene ontology terms. PLoS One, 6(7), e21800. 21789182 10.1371/journal.pone.0021800 PMC3138752

[age13490-bib-0033] Taylor, T.H. , Gitlin, S.A. , Patrick, J.L. , Crain, J.L. , Wilson, J.M. & Griffin, D.K. (2014) The origin, mechanisms, incidence and clinical consequences of chromosomal mosaicism in humans. Human Reproduction Update, 20(4), 571–581. 24667481 10.1093/humupd/dmu016

[age13490-bib-0034] Truszkowska, G.T. , Bilińska, Z.T. , Muchowicz, A. , Pollak, A. , Biernacka, A. , Kozar‐Kamińska, K. et al. (2017) Homozygous truncating mutation in NRAP gene identified by whole exome sequencing in a patient with dilated cardiomyopathy. Scientific Reports, 7(1), 3362. 28611399 10.1038/s41598-017-03189-8 PMC5469774

[age13490-bib-0035] Vogel, B. , Wagner, H. , Gmoser, J. , Wörner, A. , Löschberger, A. , Peters, L. et al. (2016) Touch‐free measurement of body temperature using close‐up thermography of the ocular surface. MethodsX, 3, 407–416. 27284532 10.1016/j.mex.2016.05.002 PMC4887592

[age13490-bib-0037] Waterhouse, A. , Bertoni, M. , Bienert, S. , Studer, G. , Tauriello, G. , Gumienny, R. et al. (2018) SWISS‐MODEL: homology modelling of protein structures and complexes. Nucleic Acids Research, 46(W1), W296–W303. Available from: 10.1093/nar/gky427 29788355 PMC6030848

[age13490-bib-0038] Wen, Y. & Crowley, S.D. (2018) Renal effects of cytokines in hypertension. Current Opinion in Nephrology and Hypertension, 27(2), 70–76. 29140820 10.1097/MNH.0000000000000385 PMC5792304

[age13490-bib-0039] Wu, H. , Guang, X. , Al‐Fageeh, M.B. , Cao, J. , Pan, S. , Zhou, H. et al. (2014) Camelid genomes reveal evolution and adaptation to desert environments. Nature Communications, 5, 5188. 10.1038/ncomms6188 25333821

