# Supplementary material for: Exploring the importance of predicted camel NRAP exon 4 for environmental adaptation using a mouse model
Source: Anim Genet. 2024 Oct 30;56(1):e13490. doi: 10.1111/age.13490 (PMC11666923; doi:10.1111/age.13490)
Supplement: Supplementary file 1 — Figure S1. Figure S2. Figure S3. Figure S4. [file AGE-56-0-s002.pdf]

(a)

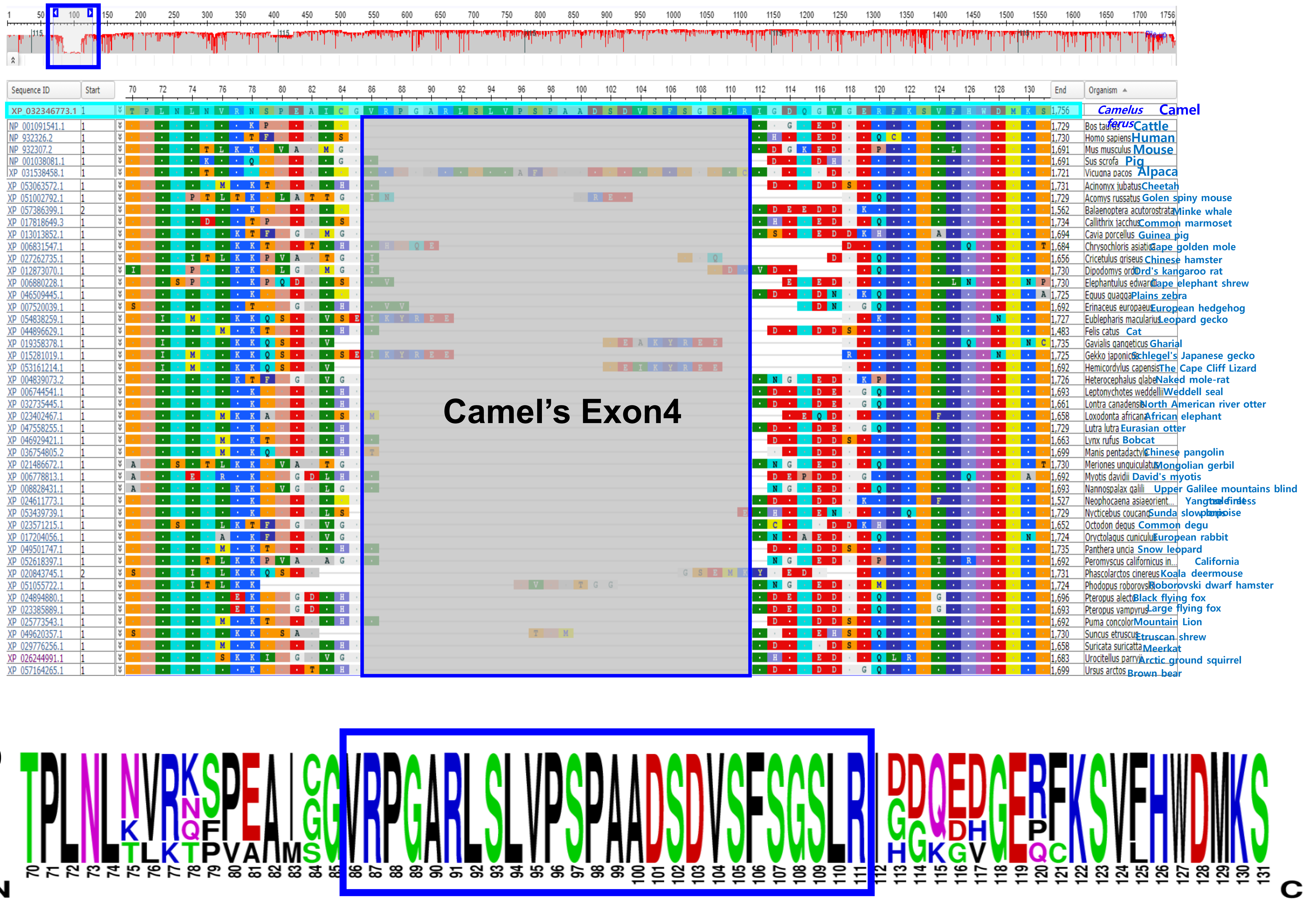

Sequence ID
Start
End
Organism
Camelus ferus Camel
Bos taurus Cattle
Homo sapiens Human
Mus musculus Mouse
Sus scrofa Pig
Vicugna pacos Alpaca
Acinonyx jubatus Cheetah
Acomys russatus Golden spiny mouse
Balaenoptera acutorostrata Minke whale
Callithrix jacchus Common marmoset
Cavia porcellus Guinea pig
Chrysochloris asiatica Cape golden mole
Cricetulus griseus Chinese hamster
Dipodomys ordii Ord's kangaroo rat
Elephantulus edwardii Cape elephant shrew
Equus quagga Plains zebra
Erinaceus europaeus European hedgehog
Eublepharis macularius Leopard gecko
Felis catus Cat
Gavialis gangeticus Gharial
Gekko japonicus Schlegel's Japanese gecko
Hemicordylus capensis The Cape Cliff Lizard
Heterocephalus glaber Naked mole-rat
Leptonychotes weddellii Weddell seal
Lontra canadensis North American river otter
Loxodonta africana African elephant
Lutra lutra Eurasian otter
Lynx rufus Bobcat
Manis pentadactyla Chinese pangolin
Meriones unguiculatus Mongolian gerbil
Myotis davidii David's myotis
Nannospalax galili Upper Galilee mountains blind mole rat
Neophocaena asiaeorientalis Yangtze finless porpoise
Nycticebus coucang Sunda slow loris
Octodon degus Common degu
Oryctolagus cuniculus European rabbit
Panthera uncia Snow leopard
Peromyscus californicus California deermouse
Phascolarctos cinereus Koala
Phodopus roborovskii Roborovski dwarf hamster
Pteropus alecto Black flying fox
Pteropus vampyrus Large flying fox
Puma concolor Mountain Lion
Suncus etruscus Etruscan shrew
Suricata suricatta Meerkat
Urocitellus parryii Arctic ground squirrel
Ursus arctos Brown bear
Camel's Exon4
N
C

(b)

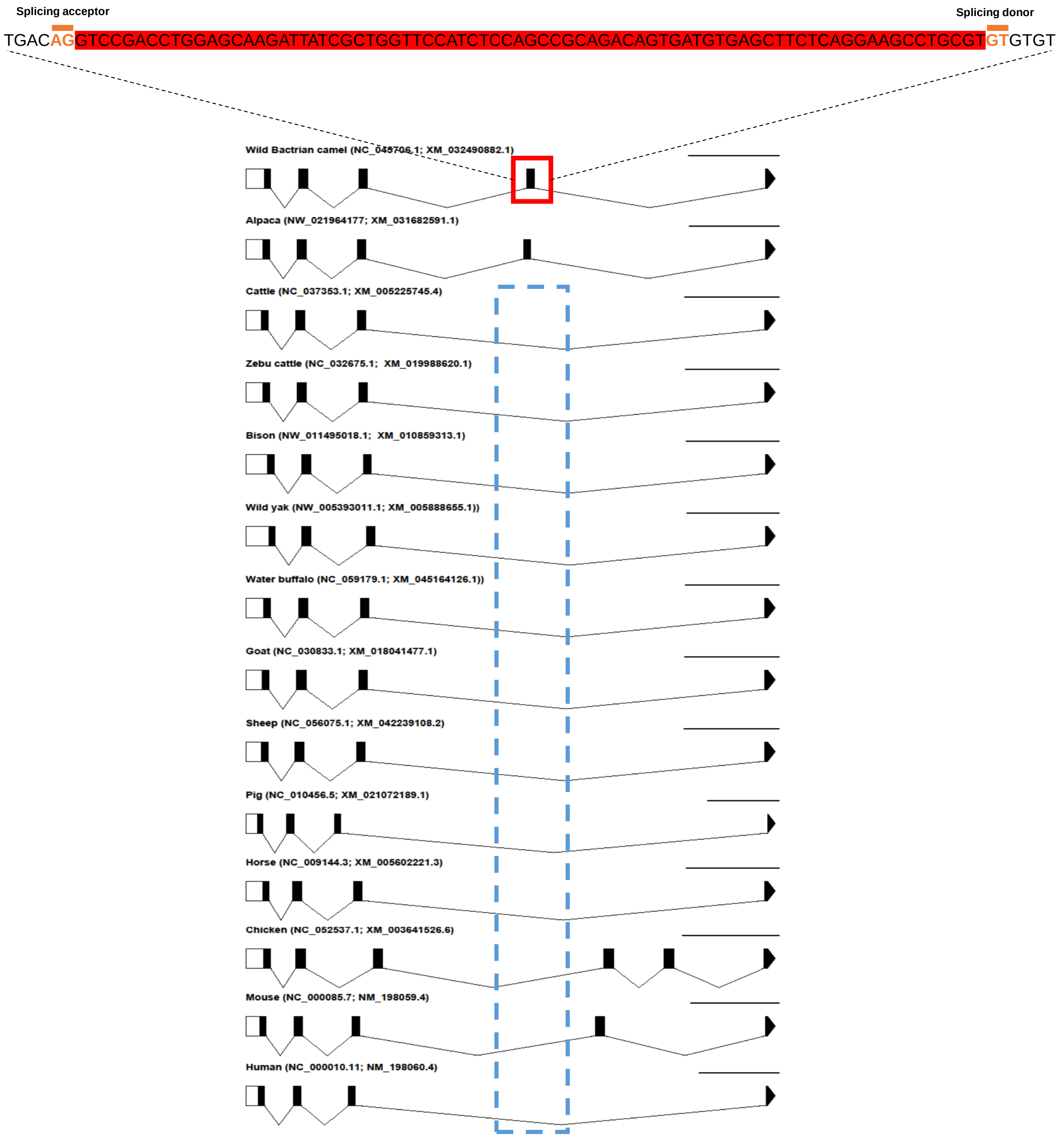
Splicing acceptor
Splicing donor
TGACAGGTCCGACCTGGAGCAAGATTATCGCTGGTTCCATCTCCAGCCGCAGACAGTGATGTGAGCTTCTCAGGAAGCCTGCGTGTGTGT
Wild Bactrian camel (NC_045706.1; XM_032490882.1)
Alpaca (NW_021964177; XM_031682591.1)
Cattle (NC_037353.1; XM_005225745.4)
Zebu cattle (NC_032675.1; XM_019988620.1)
Bison (NW_011495018.1; XM_010859313.1)
Wild yak (NW_005393011.1; XM_005888655.1))
Water buffalo (NC_059179.1; XM_045164126.1))
Goat (NC_030833.1; XM_018041477.1)
Sheep (NC_056075.1; XM_042239108.2)
Pig (NC_010456.5; XM_021072189.1)
Horse (NC_009144.3; XM_005602221.3)
Chicken (NC_052537.1; XM_003641526.6)
Mouse (NC_000085.7; NM_198059.4)
Human (NC_000010.11; NM_198060.4)

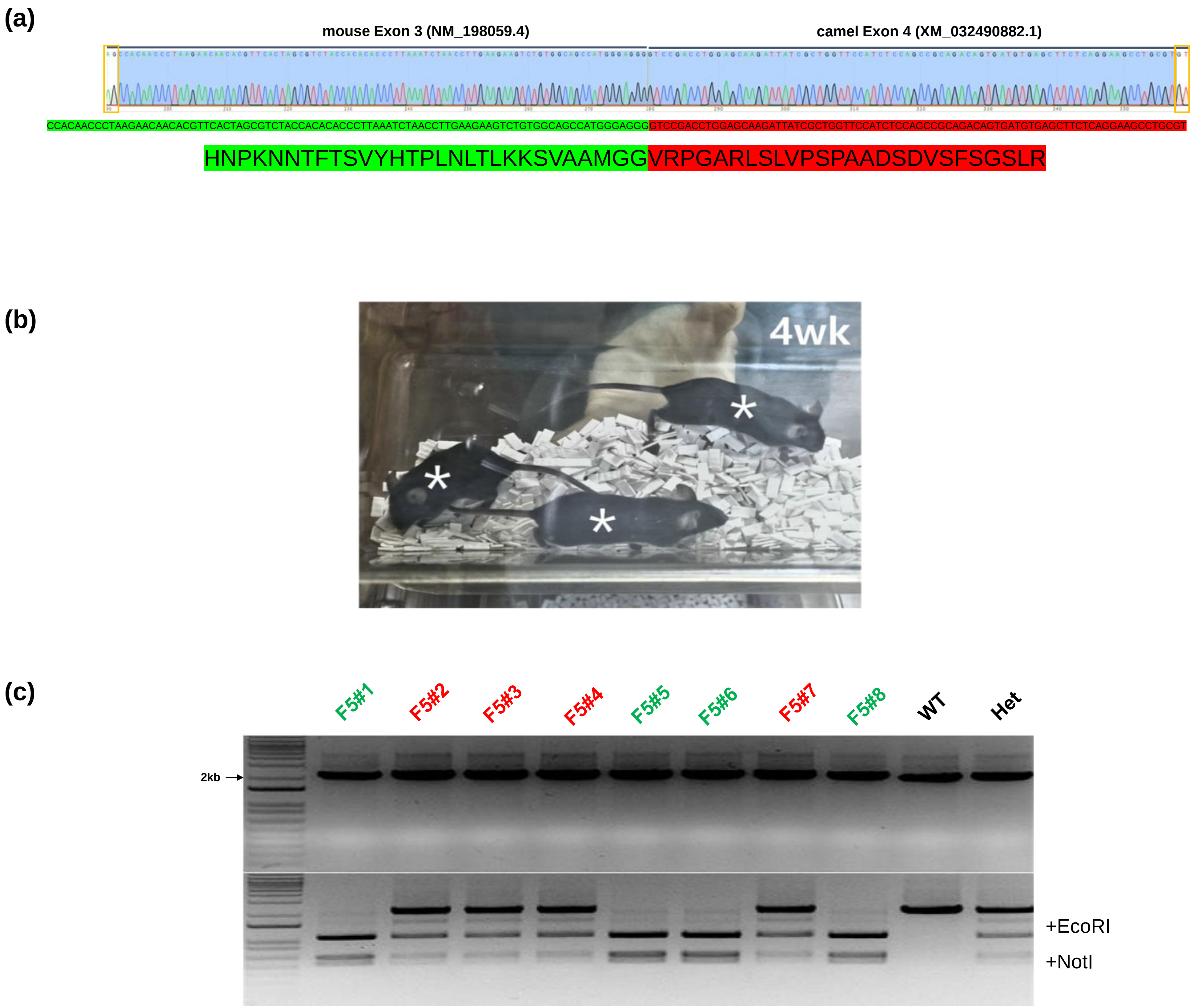

(a)
mouse Exon 3 (NM_198059.4)
camel Exon 4 (XM_032490882.1)
CCACAACCCTAAGAACAACACGTTCACTAGCGTCTACCACACACCCTTAAATCTAACCTTGAAGAAGTCTGTGGCAGCCATGGGAGGGGTCCGACCTGGAGCAAGATTATCGCTGGTTCCATCTCCAGCCGCAGACAGTGATGTGAGCTTCTCAGGAAGCCTGCGT
HNPKNNTFTSVYHTPLNLTLKKSVAAMGGVRPGARLSLVPSPAADSDVSFSGSLR
(b)
4wk
(c)
F5#1
F5#2
F5#3
F5#4
F5#5
F5#6
F5#7
F5#8
WT
Het
2kb
+EcoRI
+NotI

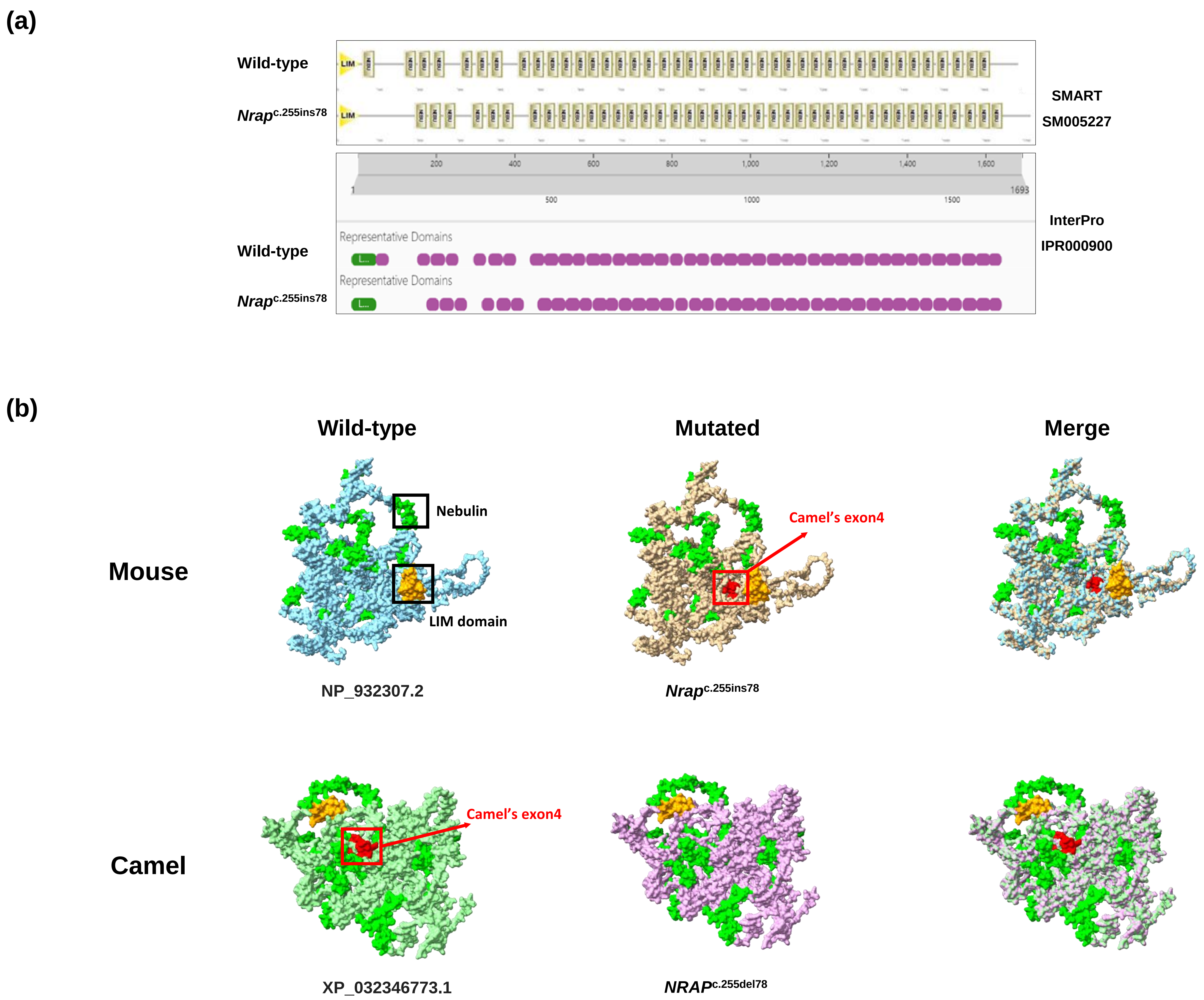

(a)
Wild-type
Nrap c.255ins78
SMART
SM005227
Representative Domains
Wild-type
Nrap c.255ins78
InterPro
IPR000900
(b)
Wild-type
Mutated
Merge
Mouse
Nebulin
LIM domain
Camel's exon4
NP_932307.2
Nrap c.255ins78
Camel
Camel's exon4
XP_032346773.1
NRAP c.255del78
